# Supplementary material for: From sequence to enzyme mechanism using multi-label machine learning
Source: BMC Bioinformatics. 2014 May 19;15:150. doi: 10.1186/1471-2105-15-150 (PMC4229970; doi:10.1186/1471-2105-15-150)
Supplement: Additional file 2 — Java code of ml2db. Additional file ml2db_code.tar.gz contains the Java source code to run the multi-label machine learning experiments and save the results to database. The code’s Javadoc is included. [file 1471-2105-15-150-S2.zip › additional file 2/ml2db/utils/doc/uk/ac/standrews/utils/main/collectionutils/ArrayUtils.html]

ArrayUtils


JavaScript is disabled on your browser.


- Overview
- Package
- Class
- Use
- Tree
- Deprecated
- Index
- Help

- Prev Class
- Next Class

- Frames
- No Frames

- All Classes

- Summary:
- Nested |
- Field |
- Constr |
- Method

- Detail:
- Field |
- Constr |
- Method


uk.ac.standrews.utils.main.collectionutils

## Class ArrayUtils

- java.lang.Object
- - uk.ac.standrews.utils.main.collectionutils.ArrayUtils

- ---

    

  ```
  public class ArrayUtils
  extends java.lang.Object
  ```

  Utilities to manipulate arrays. Reminder: indexing of array of arrays cells >
  [row index][column index]

  Version:
  :   4 Jan 2008

  Author:
  :   Luna De Ferrari luna.deferrari-at-ed.ac.uk

- - ### Constructor Summary

    Constructors

    Constructor and Description

    `ArrayUtils()`
  - ### Method Summary

    Methods

    | Modifier and Type | Method and Description |
    | --- | --- |
    | `static boolean` | `arrayIsEmpty(java.lang.String[] array)` Checks whether an array is empty |
    | `static double` | `averageArray(double[] array)` |
    | `static java.lang.String` | `booleanArrayToString(boolean[] array)` |
    | `static java.lang.String` | `doubleArrayToString(double[] array)` Prints an array of objects |
    | `static java.util.Vector<java.lang.String>` | `fromArrayofArraysToColumnVector(java.lang.String[][] array, int columnIndex)` Converts a column of an array of arrays into a vector |
    | `static java.util.ArrayList` | `fromArrayToArrayList(java.lang.Object[] array)` Creates an arrayList from an array |
    | `static java.lang.String` | `fromArrayToString(boolean[] array)` |
    | `static java.lang.String` | `fromArrayToString(java.lang.Object[] array)` From an array of objects to a string. |
    | `static java.util.Vector<java.lang.Object>` | `fromArrayToVector(java.lang.Object[] array)` Creates a vector from an array |
    | `static java.util.ArrayList<java.lang.String>` | `fromStringArrayToArrayList(java.lang.String[] array)` Creates an arrayList from an array |
    | `static int[]` | `getCommonElements(int[] list1, int[] list2)` Gets the elements present in both lists |
    | `static java.util.LinkedHashSet` | `getCommonElements(java.lang.Object[] list1, java.lang.Object[] list2)` Gets the elements present in both lists |
    | `static java.lang.String` | `getString(double[] array)` Returns a string representation for an array |
    | `static java.lang.String` | `getString(int[] array)` Returns a string representation for an array |
    | `static java.lang.String` | `getString(java.lang.String[] array)` Returns a string representation for an array |
    | `static java.lang.String` | `getTabSeparatedString(int[] array, java.lang.String separator)` Returns a separator separated string representation for an array |
    | `static void` | `printArray(java.lang.Object[] array)` Prints an array of objects |
    | `static void` | `printArray(java.lang.String[] array)` Prints an array (String[]) |
    | `static void` | `printArrayOfArrays(double[][] matrix)` Prints an array of arrays (String[][]) |
    | `static void` | `printArrayOfArrays(java.lang.String[][] matrix)` Prints an array of arrays (String[][]) |
    | `static java.lang.String[][]` | `stringToArrayOfArrays(java.lang.String string, java.lang.String regExp)` Reads a special-character-separated string (eg comma or tab separated) and creates an array of rows-columns. |
    | `static double` | `sumArray(double[] array)` |

    - ### Methods inherited from class java.lang.Object

      `equals, getClass, hashCode, notify, notifyAll, toString, wait, wait, wait`

- - ### Constructor Detail


    - #### ArrayUtils

      ```
      public ArrayUtils()
      ```
  - ### Method Detail


    - #### arrayIsEmpty

      ```
      public static boolean arrayIsEmpty(java.lang.String[] array)
      ```

      Checks whether an array is empty

      Parameters:
      :   `array` - an array of strings (String[])

      Returns:
      :   true if the array is empty, false otherwise


    - #### averageArray

      ```
      public static double averageArray(double[] array)
      ```


    - #### doubleArrayToString

      ```
      public static java.lang.String doubleArrayToString(double[] array)
      ```

      Prints an array of objects

      Parameters:
      :   `array` -


    - #### fromArrayofArraysToColumnVector

      ```
      public static java.util.Vector<java.lang.String> fromArrayofArraysToColumnVector(java.lang.String[][] array,
                                                                       int columnIndex)
      ```

      Converts a column of an array of arrays into a vector

      Parameters:
      :   `array` - an array of arrays
      :   `columnIndex` - the column index (the first column index is 0)

      Returns:
      :   a vector containing the first column


    - #### fromArrayToArrayList

      ```
      public static java.util.ArrayList fromArrayToArrayList(java.lang.Object[] array)
      ```

      Creates an arrayList from an array

      Parameters:
      :   `array` - a Object[]

      Returns:
      :   an ArrayList


    - #### fromArrayToString

      ```
      public static java.lang.String fromArrayToString(java.lang.Object[] array)
      ```

      From an array of objects to a string.

      Parameters:
      :   `array` -


    - #### fromArrayToString

      ```
      public static java.lang.String fromArrayToString(boolean[] array)
      ```


    - #### fromArrayToVector

      ```
      public static java.util.Vector<java.lang.Object> fromArrayToVector(java.lang.Object[] array)
      ```

      Creates a vector from an array

      Parameters:
      :   `array` - a Object[]

      Returns:
      :   a vector


    - #### fromStringArrayToArrayList

      ```
      public static java.util.ArrayList<java.lang.String> fromStringArrayToArrayList(java.lang.String[] array)
      ```

      Creates an arrayList from an array

      Parameters:
      :   `array` - a String[]

      Returns:
      :   an ArrayList


    - #### getCommonElements

      ```
      public static int[] getCommonElements(int[] list1,
                            int[] list2)
      ```

      Gets the elements present in both lists

      Parameters:
      :   `list1` - a list
      :   `list2` - another list

      Returns:
      :   the set containing the common elements (each element only once)


    - #### getCommonElements

      ```
      public static java.util.LinkedHashSet getCommonElements(java.lang.Object[] list1,
                                              java.lang.Object[] list2)
      ```

      Gets the elements present in both lists

      Parameters:
      :   `list1` - a list
      :   `list2` - another list

      Returns:
      :   the set containing the common elements (each element only once)


    - #### getString

      ```
      public static java.lang.String getString(double[] array)
      ```

      Returns a string representation for an array

      Parameters:
      :   `array` - the String array

      Returns:
      :   a string representation for the array


    - #### getString

      ```
      public static java.lang.String getString(int[] array)
      ```

      Returns a string representation for an array

      Parameters:
      :   `array` - the String array

      Returns:
      :   a string representation for the array


    - #### getString

      ```
      public static java.lang.String getString(java.lang.String[] array)
      ```

      Returns a string representation for an array

      Parameters:
      :   `array` - the String array

      Returns:
      :   a string representation for the array


    - #### getTabSeparatedString

      ```
      public static java.lang.String getTabSeparatedString(int[] array,
                                           java.lang.String separator)
      ```

      Returns a separator separated string representation for an array

      Parameters:
      :   `array` - the String array

      Returns:
      :   a separator separated string representation for the array


    - #### printArray

      ```
      public static void printArray(java.lang.Object[] array)
      ```

      Prints an array of objects

      Parameters:
      :   `array` -


    - #### printArray

      ```
      public static void printArray(java.lang.String[] array)
      ```

      Prints an array (String[])

      Parameters:
      :   `array` - the array to be printed (String[])


    - #### printArrayOfArrays

      ```
      public static void printArrayOfArrays(double[][] matrix)
      ```

      Prints an array of arrays (String[][])

      Parameters:
      :   `matrix` - the 2 dimentional array to be printed (String[row][column])


    - #### printArrayOfArrays

      ```
      public static void printArrayOfArrays(java.lang.String[][] matrix)
      ```

      Prints an array of arrays (String[][])

      Parameters:
      :   `matrix` - the 2 dimentional array to be printed (String[row][column])


    - #### stringToArrayOfArrays

      ```
      public static java.lang.String[][] stringToArrayOfArrays(java.lang.String string,
                                               java.lang.String regExp)
      ```

      Reads a special-character-separated string (eg comma or tab separated)
      and creates an array of rows-columns. Each column contains the column
      data elements Eg. for reading emboss fuzzpro format: SeqName Start End
      Score Strand Pattern\_name Mismatch DHET\_GLUOX 214 215 2 + AE . DHET\_GLUOX
      227 228 2 + AE .

      Parameters:
      :   `string` - the string to be read
      :   `regExp` - the regular expression separating the tokens in each line
          (e.g. a comma for comma separated values files)

      Returns:
      :   an array of arrays in which each element is a token from the
          string (row by columns)


    - #### sumArray

      ```
      public static double sumArray(double[] array)
      ```


    - #### booleanArrayToString

      ```
      public static java.lang.String booleanArrayToString(boolean[] array)
      ```


- Overview
- Package
- Class
- Use
- Tree
- Deprecated
- Index
- Help

- Prev Class
- Next Class

- Frames
- No Frames

- All Classes

- Summary:
- Nested |
- Field |
- Constr |
- Method

- Detail:
- Field |
- Constr |
- Method
